# 9,10-Dihydr­oxy-4,4-dimethyl-5,8-dihydro­anthracen-1(4*H*)-one

**DOI:** 10.1107/S1600536808010891

**Published:** 2008-06-21

**Authors:** Oney Ramírez-Rodríguez, Maximiliano Martínez-Cifuentes, Andres Ibañez, Andrés Vega, Ramiro Araya-Maturana

**Affiliations:** a Departamento de Química Orgánica, Facultad de Ciencias Químicas y Farmaceúticas, Universidad de Chile, Casilla 233, Santiago, Chile; b Centro de Investigación Interdisciplinaria Avanzada en Ciencia de los Materiales, CIMAT, Universidad de Chile, Santiago, Chile; c Laboratorio de Recursos Naturales, Departamento de Ciencias Químicas, Facultad de Ecología y Recursos Naturales, Universidad Andrés Bello, Av. República 275, Santiago, Chile

## Abstract

In the title mol­ecule, C_16_H_16_O_3_, the ring system is planar and an intramolecular hydrogen bond is present. The mol­ecular packing is dominated by an inter­molecular hydrogen bond and by π-stacking inter­actions [inter­planar separation 3.8012 Å].

## Related literature

For related literature, see: Allen (2002 ▶); Araya-Maturana *et al.* (2006 ▶, 2007 ▶); Desiraju (2002 ▶); Joshi *et al.* (1997 ▶); Valderrama *et al.* (1993 ▶).
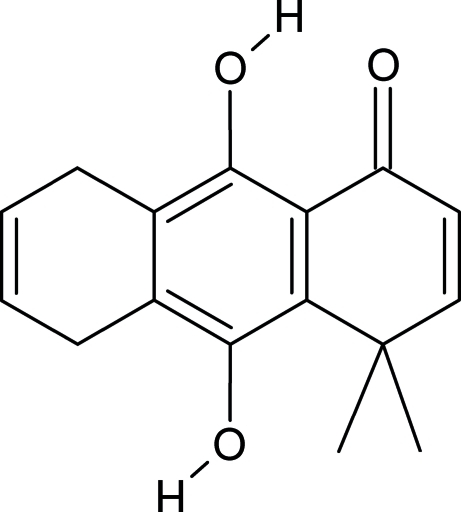


## Experimental

### 

#### Crystal data

C_16_H_16_O_3_
*M* *_r_* = 256.29
Orthorhombic, 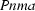

*a* = 8.5944 (5) Å
*b* = 7.6024 (5) Å
*c* = 19.2949 (12) Å
*V* = 1260.69 (14) Å^3^
*Z* = 4
Mo *K*α radiation
μ = 0.09 mm^−1^
*T* = 150 (2) K
0.43 × 0.38 × 0.08 mm

#### Data collection

Siemens SMART CCD area-detector diffractometer
Absorption correction: multi-scan (*SADABS* in *SAINT-NT*; Bruker, 1999 ▶) *T*_min_ = 0.961, *T*_max_ = 0.993
7422 measured reflections
1198 independent reflections
948 reflections with *I* > 2σ(*I*)
*R*_int_ = 0.043

#### Refinement

*R*[*F* ^2^ > 2σ(*F* ^2^)] = 0.044
*wR*(*F* ^2^) = 0.118
*S* = 1.01
1198 reflections
112 parameters
H-atom parameters constrained
Δρ_max_ = 0.33 e Å^−3^
Δρ_min_ = −0.29 e Å^−3^

### 

Data collection: *SMART-NT* (Bruker, 2001 ▶); cell refinement: *SAINT-NT* (Bruker, 1999 ▶); data reduction: *SAINT-NT*; program(s) used to solve structure: *SHELXTL-NT* (Sheldrick, 2008 ▶); program(s) used to refine structure: *SHELXTL-NT*; molecular graphics: *SHELXTL-NT*; software used to prepare material for publication: *SHELXTL-NT*.

## Supplementary Material

Crystal structure: contains datablocks I, global. DOI: 10.1107/S1600536808010891/hg2395sup1.cif

Structure factors: contains datablocks I. DOI: 10.1107/S1600536808010891/hg2395Isup2.hkl

Additional supplementary materials:  crystallographic information; 3D view; checkCIF report

## Figures and Tables

**Table 1:** Hydrogen-bond geometry (Å, °)

*D*—H⋯*A*	*D*—H	H⋯*A*	*D*⋯*A*	*D*—H⋯*A*
O2—H2⋯O1	0.84	1.77	2.5172 (18)	147
O3—H3⋯O1^i^	0.84	2.03	2.8022 (18)	152

Symmetry code: (i) 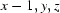.

**Fig. 1.**
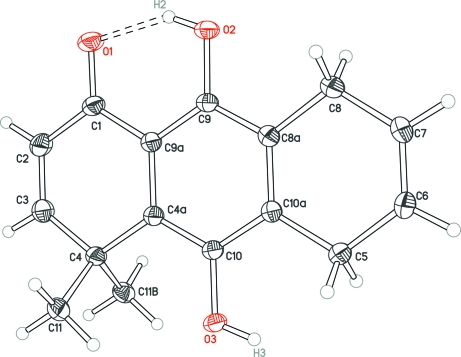
Molecular structure diagramas for I showing atom numbering scheme. Displacement ellipsoids are at 33% probability level and H atoms are shown as spheres of arbitrary radii. Letter a corresponds to the names of fused carbon atoms according to the nomenclature rules. Letter B to symmcode x, -y + 1/2, z.

**Fig. 2.**
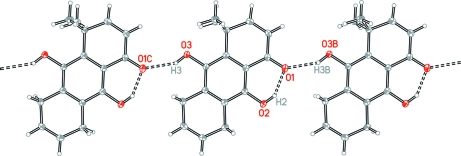
Molecular packing view for I along [010]. [symmetry codes: (B) x + 1, y, z; (C) x - 1, y, z].

**Fig. 3.**
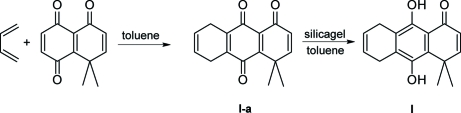
The formation of the title compound.

## supplementary crystallographic information

### Comment

The title hydroquinone I is a potent antioxidant (Araya-Maturana *et al*, 2007) and respiration inhibitor of cancer cells (Araya-Maturana *et al.*, 2006). In mouse mammary adenocarcinoma TA3 and their multidrug-resistant variant TA3-MTX-*R* lines exhibit IC_50_ values below 10^-4^M. Moreover, this compound inhibits the growth of the human tumor U937 cell line at low micromolar concentrations (Araya-Maturana *et al.*, 2006).

The molecule consists of three six-membered carbon rings fused trough atoms 4a, 9a in a side and trough carbons 8a, 10a in the other, to give rise an anthracene skeleton, substituted with an oxo, a *gem*-dimethyl and two hydroxyl groups at positions 1, 4, 9 and 10 respectively, as shown in Scheme 1. The central ring is aromatic, and double bonds are also found between carbons 2 - 3 and 6 - 7. This double bonds distribution leads to the core to be strictly planar. In fact, all the carbon atoms in the skeleton lies on the crystallographic mirror plane *m* from space group P*nma*. The same happens with the oxo oxygen atom O1 and the hydroxyl groups O2 and O3. Interestingly, the hydroxyl hydrogen atoms H2 and H3 display a *trans* correlation. The planarity of the molecule together with the proximity of the oxo oxygen atom (O1) and the hydroxyl hydrogen atom (H2) leads to the presence of an intramolecular O—H···O hydrogen bond with O···O of 2.5172 (18) Å, suggesting a rather strong bond (Desiraju, 2002), which is present still in CDCl_3_ solution, as indicated by NMR (Araya-Maturana *et al*, 2007). Few structures with this or some closely related pattern of substitution could be found in Cambridge Structural database (v 5.29, Allen, 2002), being 1,4-Dihydro-9,10-anthrahydroquinone the best, probably the one to the best of our knowledge, example (Joshi *et al.*, 1997).

The molecular packing is also dominated by the hydrogen bond, this time between vicinal molecules. As depicted in Figure 2, a planar chain is produced by means of the interacion of the "terminal" hydroxyl hydrogen atom H3 with the oxo oxygen O1 from the nearest molecule (*x* - 1, *y*, *z*), in a "head to tail" arrangement in the [100] direction. The O···O distance, 2.8022 (18) Å, suggest a weaker interaction. Layers of molecules are defined in the packing by putting this chains one together the other, with no strong interaction between them. Any of the chain is contained in the *x*, 1/4, *z* plane. The next layer, *x*, 3/4, *z* is separated from the first in b/2, 3.8012 Å, a typical value for the aromatic π-stacking interaction.

### Experimental

The molecule was synthesized by the Diels–Alder reaction between 8,8-dimethylnaphthalene-1,4,5(8*H*)-trione and butadiene). The cycloaddition takes place exclusively at external quinone doble bond affording the corresponding adduct I-a (See Scheme 2). Enolization of the adduct I-a with silicagel in toluene yield the hydroquinone I. (Valderrama *et al.*, 1993). The ^1^H-NMR spectrum in CDCl_3_ of I exhibits a sharp singlet at 13.08 p.p.m. indicating that hydrogen bonding is also present in solution. This characteristic is important regarding antitumor and antioxidant properties.

### Refinement

The hydrogen atoms positions were calculated after each cycle of refinement with *SHELXL* (Bruker,1999) using a riding model for each structure, with C—H distances in the range 0.95 to 0.99 Å and O—H equal to 0.84 Å. *U*_iso_(H) values were set equal to 1.5*U*_eq_ of the parent carbon atom for methyl groups and hydroxyl hydrogen atoms, while 1.2*U*_eq_ for the others.

### Crystal data

**Table:**

C_16_H_16_O_3_	*F*_000_ = 544
*M**_r_* = 256.29	*D*_x_ = 1.350 Mg m^−^^3^
Orthorhombic, *P**n**m**a*	Mo *K*α radiation λ = 0.71073 Å
Hall symbol: -P 2ac 2n	Cell parameters from 2361 reflections
*a* = 8.5944 (5) Å	θ = 2.6–25.0º
*b* = 7.6024 (5) Å	µ = 0.09 mm^−^^1^
*c* = 19.2949 (12) Å	*T* = 150 (2) K
*V* = 1260.69 (14) Å^3^	Plate, orange
*Z* = 4	0.43 × 0.38 × 0.08 mm

### Data collection

**Table:**

Siemens SMART CCD area-detector diffractometer	1198 independent reflections
Radiation source: fine-focus sealed tube	948 reflections with *I* > 2σ(*I*)
Monochromator: graphite	*R*_int_ = 0.043
*T* = 150(2) K	θ_max_ = 25.0º
φ and ω scans	θ_min_ = 2.1º
Absorption correction: multi-scan(SADABS in SAINT-NT; Bruker, 1999)	*h* = −10→10
*T*_min_ = 0.961, *T*_max_ = 0.993	*k* = −9→9
7422 measured reflections	*l* = −22→22

### Refinement

**Table:**

Refinement on *F*^2^	Secondary atom site location: difference Fourier map
Least-squares matrix: full	Hydrogen site location: inferred from neighbouring sites
*R*[*F*^2^ > 2σ(*F*^2^)] = 0.044	H-atom parameters constrained
*wR*(*F*^2^) = 0.118	*w* = 1/[σ^2^(*F*_o_^2^) + (0.0796*P*)^2^] where *P* = (*F*_o_^2^ + 2*F*_c_^2^)/3
*S* = 1.01	(Δ/σ)_max_ < 0.001
1198 reflections	Δρ_max_ = 0.33 e Å^−^^3^
112 parameters	Δρ_min_ = −0.29 e Å^−^^3^
Primary atom site location: structure-invariant direct methods	Extinction correction: none

### Special details

**Table:**

Experimental. 10 s by frame separated by 0.3 °
Geometry. All e.s.d.'s (except the e.s.d. in the dihedral angle between two l.s. planes) are estimated using the full covariance matrix. The cell e.s.d.'s are taken into account individually in the estimation of e.s.d.'s in distances, angles and torsion angles; correlations between e.s.d.'s in cell parameters are only used when they are defined by crystal symmetry. An approximate (isotropic) treatment of cell e.s.d.'s is used for estimating e.s.d.'s involving l.s. planes.
Refinement. Refinement of *F*^2^ against ALL reflections. The weighted *R*-factor *wR* and goodness of fit *S* are based on *F*^2^, conventional *R*-factors *R* are based on *F*, with *F* set to zero for negative *F*^2^. The threshold expression of *F*^2^ > σ(*F*^2^) is used only for calculating *R*-factors(gt) *etc*. and is not relevant to the choice of reflections for refinement. *R*-factors based on *F*^2^ are statistically about twice as large as those based on *F*, and *R*- factors based on ALL data will be even larger.

### Fractional atomic coordinates and isotropic or equivalent isotropic displacement parameters (Å^2^)

**Table:**

	*x*	*y*	*z*	*U*_iso_*/*U*_eq_	Occ. (<1)
O1	1.23661 (15)	0.2500	0.97450 (7)	0.0430 (4)	
C1	1.1134 (2)	0.2500	0.93987 (9)	0.0316 (5)	
C2	1.1204 (2)	0.2500	0.86508 (10)	0.0394 (5)	
H2B	1.2188	0.2500	0.8427	0.047*	
C3	0.9915 (2)	0.2500	0.82719 (10)	0.0366 (5)	
H3A	1.0040	0.2500	0.7783	0.044*	
C4	0.8283 (2)	0.2500	0.85428 (9)	0.0276 (5)	
C11	0.74913 (15)	0.41540 (18)	0.82447 (7)	0.0319 (4)	
H11A	0.7543	0.4123	0.7737	0.048*	
H11B	0.8027	0.5206	0.8415	0.048*	
H11C	0.6400	0.4184	0.8392	0.048*	
C4A	0.8227 (2)	0.2500	0.93365 (10)	0.0250 (4)	
C10	0.68158 (19)	0.2500	0.96919 (10)	0.0257 (5)	
O3	0.54736 (14)	0.2500	0.93053 (6)	0.0349 (4)	
H3	0.4700	0.2500	0.9571	0.052*	
C10A	0.67531 (19)	0.2500	1.04249 (9)	0.0246 (5)	
C5	0.5186 (2)	0.2500	1.07767 (10)	0.0293 (5)	
H5A	0.4600	0.3551	1.0623	0.035*	0.50
H5B	0.4600	0.1449	1.0623	0.035*	0.50
C6	0.5260 (2)	0.2500	1.15519 (10)	0.0302 (5)	
H6A	0.4305	0.2500	1.1800	0.036*	
C7	0.6566 (2)	0.2500	1.19124 (10)	0.0299 (5)	
H7A	0.6495	0.2500	1.2404	0.036*	
C8	0.8143 (2)	0.2500	1.15932 (10)	0.0311 (5)	
H8A	0.8718	0.1448	1.1755	0.037*	0.50
H8B	0.8718	0.3552	1.1755	0.037*	0.50
C8A	0.8116 (2)	0.2500	1.08099 (9)	0.0252 (4)	
C9	0.9537 (2)	0.2500	1.04619 (9)	0.0257 (5)	
O2	1.08404 (15)	0.2500	1.08586 (6)	0.0356 (4)	
H2	1.1629	0.2500	1.0602	0.053*	
C9A	0.9614 (2)	0.2500	0.97343 (10)	0.0255 (5)	

### Atomic displacement parameters (Å^2^)

**Table:**

	*U*^11^	*U*^22^	*U*^33^	*U*^12^	*U*^13^	*U*^23^
O1	0.0199 (7)	0.0740 (12)	0.0351 (8)	0.000	−0.0021 (6)	0.000
C1	0.0210 (10)	0.0430 (12)	0.0310 (11)	0.000	−0.0009 (8)	0.000
C2	0.0246 (10)	0.0629 (14)	0.0306 (12)	0.000	0.0075 (9)	0.000
C3	0.0322 (11)	0.0527 (13)	0.0248 (10)	0.000	0.0037 (8)	0.000
C4	0.0232 (9)	0.0384 (12)	0.0213 (10)	0.000	−0.0015 (7)	0.000
C11	0.0346 (8)	0.0375 (8)	0.0237 (7)	−0.0025 (6)	−0.0025 (5)	0.0023 (6)
C4A	0.0230 (10)	0.0287 (10)	0.0232 (10)	0.000	−0.0008 (7)	0.000
C10	0.0211 (10)	0.0313 (11)	0.0246 (10)	0.000	−0.0020 (7)	0.000
O3	0.0189 (7)	0.0610 (10)	0.0247 (7)	0.000	−0.0024 (5)	0.000
C10A	0.0243 (10)	0.0264 (10)	0.0233 (10)	0.000	0.0004 (7)	0.000
C5	0.0226 (10)	0.0377 (11)	0.0276 (11)	0.000	0.0003 (7)	0.000
C6	0.0286 (10)	0.0335 (11)	0.0286 (10)	0.000	0.0069 (8)	0.000
C7	0.0354 (11)	0.0323 (11)	0.0221 (10)	0.000	0.0021 (8)	0.000
C8	0.0291 (10)	0.0395 (12)	0.0247 (10)	0.000	−0.0036 (8)	0.000
C8A	0.0262 (10)	0.0261 (10)	0.0233 (10)	0.000	−0.0012 (7)	0.000
C9	0.0222 (9)	0.0307 (11)	0.0243 (10)	0.000	−0.0039 (7)	0.000
O2	0.0215 (7)	0.0583 (9)	0.0271 (8)	0.000	−0.0058 (6)	0.000
C9A	0.0225 (10)	0.0291 (10)	0.0248 (10)	0.000	−0.0008 (7)	0.000

### Geometric parameters (Å, °)

**Table:**

O1—C1	1.252 (2)	O3—H3	0.8400
C1—C2	1.444 (3)	C10A—C8A	1.387 (2)
C1—C9A	1.458 (2)	C10A—C5	1.508 (2)
C2—C3	1.327 (3)	C5—C6	1.497 (3)
C2—H2B	0.9500	C5—H5A	0.9900
C3—C4	1.497 (2)	C5—H5B	0.9900
C3—H3A	0.9500	C6—C7	1.320 (3)
C4—C4A	1.532 (3)	C6—H6A	0.9500
C4—C11^i^	1.5410 (16)	C7—C8	1.489 (3)
C4—C11	1.5410 (16)	C7—H7A	0.9500
C11—H11A	0.9800	C8—C8A	1.512 (3)
C11—H11B	0.9800	C8—H8A	0.9900
C11—H11C	0.9800	C8—H8B	0.9900
C4A—C10	1.393 (2)	C8A—C9	1.394 (2)
C4A—C9A	1.418 (2)	C9—O2	1.357 (2)
C10—O3	1.374 (2)	C9—C9A	1.405 (3)
C10—C10A	1.415 (3)	O2—H2	0.8400
			
O1—C1—C2	119.87 (16)	C10—C10A—C5	118.94 (16)
O1—C1—C9A	121.38 (17)	C6—C5—C10A	114.34 (15)
C2—C1—C9A	118.75 (16)	C6—C5—H5A	108.7
C3—C2—C1	121.05 (17)	C10A—C5—H5A	108.7
C3—C2—H2B	119.5	C6—C5—H5B	108.7
C1—C2—H2B	119.5	C10A—C5—H5B	108.7
C2—C3—C4	126.13 (18)	H5A—C5—H5B	107.6
C2—C3—H3A	116.9	C7—C6—C5	124.20 (17)
C4—C3—H3A	116.9	C7—C6—H6A	117.9
C3—C4—C4A	112.24 (15)	C5—C6—H6A	117.9
C3—C4—C11^i^	106.46 (10)	C6—C7—C8	123.79 (17)
C4A—C4—C11^i^	111.05 (10)	C6—C7—H7A	118.1
C3—C4—C11	106.46 (10)	C8—C7—H7A	118.1
C4A—C4—C11	111.05 (10)	C7—C8—C8A	113.53 (15)
C11^i^—C4—C11	109.37 (14)	C7—C8—H8A	108.9
C4—C11—H11A	109.5	C8A—C8—H8A	108.9
C4—C11—H11B	109.5	C7—C8—H8B	108.9
H11A—C11—H11B	109.5	C8A—C8—H8B	108.9
C4—C11—H11C	109.5	H8A—C8—H8B	107.7
H11A—C11—H11C	109.5	C10A—C8A—C9	118.81 (17)
H11B—C11—H11C	109.5	C10A—C8A—C8	123.28 (16)
C10—C4A—C9A	117.74 (17)	C9—C8A—C8	117.91 (15)
C10—C4A—C4	121.29 (15)	O2—C9—C8A	116.85 (16)
C9A—C4A—C4	120.97 (15)	O2—C9—C9A	121.64 (16)
O3—C10—C4A	117.62 (16)	C8A—C9—C9A	121.50 (16)
O3—C10—C10A	120.71 (15)	C9—O2—H2	109.5
C4A—C10—C10A	121.67 (16)	C9—C9A—C4A	120.08 (16)
C10—O3—H3	109.5	C9—C9A—C1	119.07 (16)
C8A—C10A—C10	120.20 (16)	C4A—C9A—C1	120.86 (16)
C8A—C10A—C5	120.86 (17)		
			
C2—C3—C4—C11	121.70 (9)	C11—C4—C4A—C9A	−119.04 (10)
C11—C4—C4A—C10	60.96 (10)		

Symmetry codes: (i) *x*, −*y*+1/2, *z*.

### Hydrogen-bond geometry (Å, °)

**Table:**

*D*—H···*A*	*D*—H	H···*A*	*D*···*A*	*D*—H···*A*
O2—H2···O1	0.84	1.77	2.5172 (18)	147
O3—H3···O1^ii^	0.84	2.03	2.8022 (18)	152

Symmetry codes: (ii) *x*−1, *y*, *z*.
